# Electrocardiogram Devices for Home Use: Technological and Clinical Scoping Review

**DOI:** 10.2196/44003

**Published:** 2023-07-07

**Authors:** Alejandra Zepeda-Echavarria, Rutger R van de Leur, Meike van Sleuwen, Rutger J Hassink, Thierry X Wildbergh, Pieter A Doevendans, Joris Jaspers, René van Es

**Affiliations:** 1 Medical Technologies and Clinical Physics Facilitation Department University Medical Center Utrecht Utrecht Netherlands; 2 Department of Cardiology Division of Heart and Lungs University Medical Center Utrecht Utrecht Netherlands; 3 Department of Cardiology Meander Medical Center Amersfoort Netherlands; 4 HeartEye BV Delft Netherlands; 5 Netherlands Heart Institute Utrecht Netherlands

**Keywords:** electrocardiogram, mobile ECG, home use ECG, wearables, medical devices, ECG clinical validation, ECG technical characteristics

## Abstract

**Background:**

Electrocardiograms (ECGs) are used by physicians to record, monitor, and diagnose the electrical activity of the heart. Recent technological advances have allowed ECG devices to move out of the clinic and into the home environment. There is a great variety of mobile ECG devices with the capabilities to be used in home environments.

**Objective:**

This scoping review aimed to provide a comprehensive overview of the current landscape of mobile ECG devices, including the technology used, intended clinical use, and available clinical evidence.

**Methods:**

We conducted a scoping review to identify studies concerning mobile ECG devices in the electronic database PubMed. Secondarily, an internet search was performed to identify other ECG devices available in the market. We summarized the devices’ technical information and usability characteristics based on manufacturer data such as datasheets and user manuals. For each device, we searched for clinical evidence on the capabilities to record heart disorders by performing individual searches in PubMed and ClinicalTrials.gov, as well as the Food and Drug Administration (FDA) 510(k) Premarket Notification and De Novo databases.

**Results:**

From the PubMed database and internet search, we identified 58 ECG devices with available manufacturer information. Technical characteristics such as shape, number of electrodes, and signal processing influence the capabilities of the devices to record cardiac disorders. Of the 58 devices, only 26 (45%) had clinical evidence available regarding their ability to detect heart disorders such as rhythm disorders, more specifically atrial fibrillation.

**Conclusions:**

ECG devices available in the market are mainly intended to be used for the detection of arrhythmias. No devices are intended to be used for the detection of other cardiac disorders. Technical and design characteristics influence the intended use of the devices and use environments. For mobile ECG devices to be intended to detect other cardiac disorders, challenges regarding signal processing and sensor characteristics should be solved to increase their detection capabilities. Devices recently released include the use of other sensors on ECG devices to increase their detection capabilities.

## Introduction

### Background

Cardiovascular diseases are the leading cause of mortality, accounting for approximately 31% of all deaths worldwide [1]. The leading contributors to cardiovascular death are ischemic heart disease, ischemic stroke, hemorrhagic stroke, hypertensive heart disease (which ultimately results in heart failure), cardiomyopathy, rheumatic heart disease, and atrial fibrillation (AF) [2]. To perform cardiovascular assessments, physicians require diagnostic tools such as the electrocardiogram (ECG) [3].

The ECG records the electrical signals generated by the heart’s electrical activity; the electrical currents arise owing to potential differences that spread to the surface of the body when cardiac impulses pass through the heart [4]. The traditional 12-lead ECG is recorded via electrodes placed on the limbs and chest wall [3]. The ECG is a tool used in the everyday practice of clinical medicine, with >300 million ECGs obtained annually [5].

The 12-lead ECG is the clinical gold standard and is reminiscent of the original recordings by Einthoven, which refers to the placement of 3 limb electrodes, from which 2 leads are measured, and other 4 leads are calculated, allowing 6 limb leads and creating a view of the heart in the vertical plane [6]. In addition, the 6 precordial leads (V1-V6) provide a view of the horizontal plane of the heart, using the Wilson central terminal as a reference [3]. Technological advances such as the miniaturization of electronic components, innovations in sensor technologies, and progress in mobile and communication technology have allowed innovations in mobile health devices. New technologies allow general practitioners or ambulance staff to record ECGs as routine in chest pain, whereas patients can perform self-monitoring at home. Thus, the ECG is moving from the clinical to the domestic environment [7].

Previous review studies on mobile ECG devices have focused primarily on wearable sensors that can be used in and outside of the clinic, the technological taxonomy of ECG devices, an analysis of single- and 3-lead devices, adhesive ECG patch devices, and devices focused only on diagnosing rhythm disorders and conduction system diseases; the studies’ secondary focus has been on the future of ECG technologies, including the necessary steps for integration in clinical infrastructures [7-13]. The published reviews have shown that a majority of mobile ECG devices are focused on screening for AF or other rhythm disorders [7,8,11,13]. The reviews partly cover the potential applications of mobile ECG devices. There is a gap in how the available devices in the market can be selected for use based on device characteristics, purpose, and clinical evidence.

### Objectives

This review aimed to provide an overview of the mobile ECG devices that are available in the market, including the technology used, their intended clinical use, and the published clinical evidence used for validation. In this review, we defined the gaps and pitfalls in commercially available and discontinued devices to provide the reader with a comprehensive overview of the current landscape of mobile ECG devices as well as their clinical purposes, clinical outcomes, and benefits. In addition, we addressed the disadvantages per type of device to highlight the most promising devices or technology used and the areas where there is room for improvement.

## Methods

### Device Searches

First, we performed a PubMed database search for ECG devices. We searched for articles published between September 6, 2012, and September 6, 2022. Article titles, keywords, and abstracts were searched using the following search terms: “Wearable Electronic Devices” (medical subject headings [MeSH] term) AND (“Electrocardiography” [MeSH term] OR “electrocardiography, ambulatory” [MeSH term]). We only included articles published in English, and the identified devices needed to be capable of recording ECGs. We excluded devices that are only capable of recording photoplethysmography.

In addition, internet searches for mobile ECG devices using the Google search engine were performed. The search words used were “electrocardiography” combined with “mobile,” “wearable,” or “handheld.” Only ECG devices were included.

### Device Characteristics

Once the mobile ECG devices were identified, we consulted their manufacturer websites to gather technical datasheets as well as user manuals and then summarized their characteristics and technical and user specifications. We also consulted the Food and Drug Administration (FDA) 510(k) Premarket Notification database and ClinicalTrials.gov to review data on devices when no data were available from the manufacturers.

**Figure 1:**
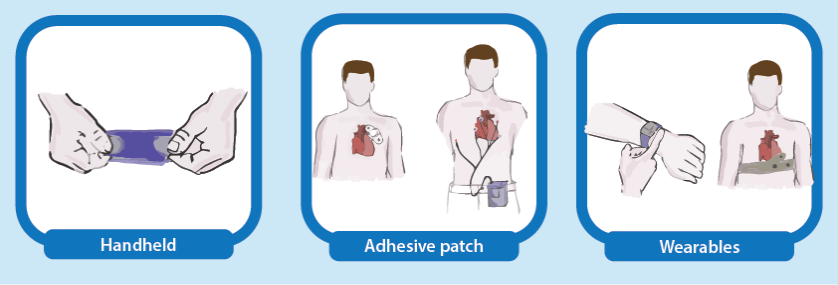
Presentation of mobile electrocardiogram device categories.

After we identified the ECG devices, based on their characteristics, all devices found were classified into three types of mobile ECG devices: (1) handheld, (2) patch, and (3) wearable (as summarized in Figure 1 and Textbox 1).

**Types of mobile electrocardiogram (ECG) devices.**

**Handheld devices**

- These devices, which have embedded dry electrodes, are required to be carried separately by users. To perform ECG recordings, users place the device on their chest or hold the device in their hands. This type of device performs ECG recordings when it is activated by users. Users perform intermittent recordings lasting <1 minute.

**Patch devices**

- These devices, which have disposable embedded electrodes or disposable surface electrodes, are usually attached to the left chest of patients. They can perform continuous recordings for up to 30 days.

**Wearables**

- These devices, which use dry metal, textile, or single-use electrodes, are used for continuous wearing during normal daily activities. Wearables are worn on the chest as a garment (eg, T-shirt), as a harness, or on the wrist as a smartwatch. Depending on the area of measurement, these devices can perform for 24 hours or obtain recordings lasting <1 minute.

For each device, we identified the intended use, recording time, and number of electrodes (instead of leads because the number of leads was not specified for some devices; it should be noted that for 3-electrode devices, 6 leads could be obtained from the calculation of the limb leads). We also detailed whether the device is stand-alone.

We registered the user characteristics (user environment, multiple areas of measurement, and setup difficulty), as well as technical characteristics (sampling rate, sampling resolution, and signal bandwidth) and compliance characteristics for use, such as the level of protection of the device against the ingress of hazardous parts and water (the ingress protection [IP] rating).

### Clinical Evidence

Finally, we searched for available clinical information by performing a search per device with the aim to identify the available clinical evidence regarding its capabilities. We identified the type of studies performed per device, whether the device had been validated for detection of certain heart conditions, and whether these studies had compared the device against 12-lead ECG devices or other mobile ECG devices.

We also analyzed the feasibility of these devices for home use while guaranteeing the safety of patients. We looked at home use compliance as well as analyzed the available clinical evidence for detection of heart disorders.

## Results

### Overview

**Figure 2:**
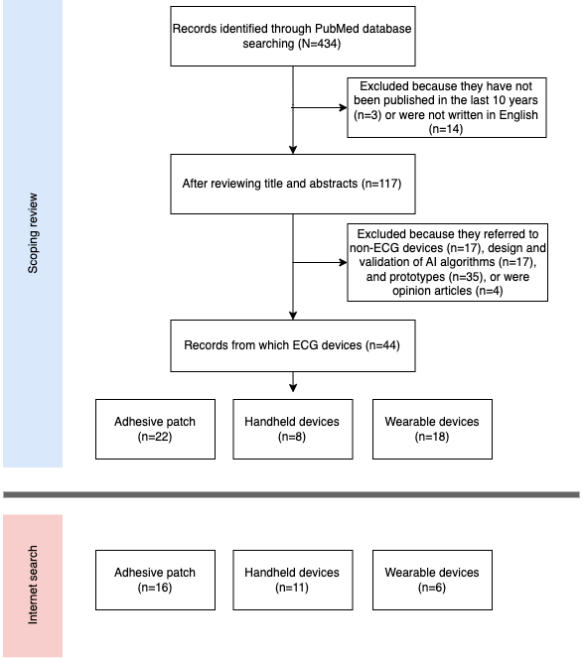
Schematic view of the methodology used for the scoping review and the internet search results. AI: artificial intelligence; ECG: electrocardiogram.

**Figure 3:**
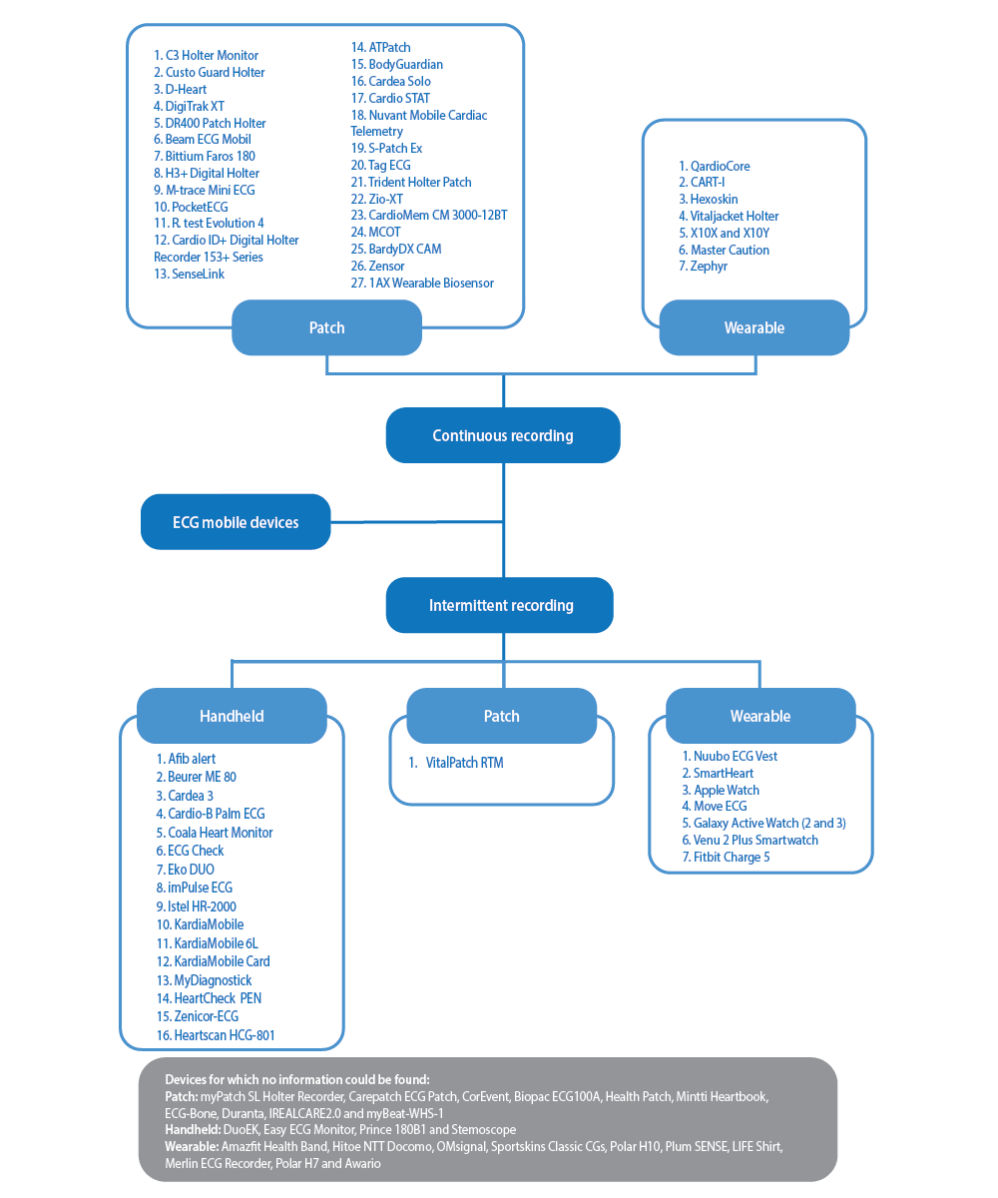
Electrocardiogram (ECG) device classification based on type of recording and type of device.

With the PubMed search, we identified 434 articles (Figure 2), of which we excluded 317 (73%) owing to publication date as well as not being written in English and after an examination of titles and abstracts, leaving 117 (27%) for analysis. Of these 117 publications, 73 (62.4%) were excluded because they referred to prototype devices (n=35, 48%), non-ECG devices (n=17, 23%), and design and validation of artificial intelligence algorithms (n=17, 23%) or were opinion articles (n=4, 5%). From the remaining articles (44/117, 37.6%), we identified 48 ECG devices, of which 22 (46%) were patch-based devices, 8 (17%) were handheld devices, and 18 (38%) were wearables. Subsequently, from the internet search, another 33 devices were identified: 16 (49%) were patch-based devices, 11 (33%) were handheld devices, and 6 (18%) were wearables. In total, 82 devices were identified for this review (Figure 2). For 58 (70%) of these 82 devices, we were able to find characteristics from manufacturer websites. We summarized and grouped the devices into continuous recording devices and intermittent recording devices (Figure 3).

### Clinical Purpose

For 3% (2/58) of the devices, the intended use information was not available from the manufacturer (Tables 1 and 2). Of the 58 devices, 21 (36%) do not state intended use regarding the detection of rhythm disorders; they are intended to be used for measuring and recording ECGs in general. However, more than half (31/58, 53%) of the devices are intended to be used when there is suspicion of arrhythmias (25/58, 43%), more specifically AF (6/58, 10%).

**Table 1:** Functionality characteristics per device (continuous recording devices).

Device		Manufacturer		Intended use	Recording time	Number of electrodes	Stand-alone	Source of clinical evaluation evidence
**Patch**								
	C3 Holter Monitor^a^		Cortrium ApS	M+R^b^	1 week	3	Yes	Clinical trials website^c^
	Custo Guard Holter^a^		Custo Med GmbH	M+R+PMD^d^	>1 day	4	Yes	—^e^
	D-Heart		D-Heart Srl	M+R	1 day	6	No	—
	DigiTrak XT		Koninklijke Philips NV	M+R+HRD^f,g^	1 week	5	Yes	Clinical trials website^c^ and PND^h^
	DR400 Patch Holter		NorthEast Monitoring Inc	ER^i^+AD^j^	>1 week	3	Yes	PND
	Beam ECG Mobil^a^		IEM GmbH	M+R+ER^g^	>1 minute	8	Yes	—
	Bittium Faros 180		Bittium	M+R+AD^g^	>1 week	2	Yes	PND
	H3+ Digital Holter^a^		Welch Allyn	M+R+AD	>1 day	5	Yes	PND
	M-trace Mini ECG^a^		M4Medical	M+R+AD^g^	1 minute	4	Yes	Clinical trials website^c^
	PocketECG^a^		Medicalgorithmics SA	M+R+AD	1 day	3	Yes	Clinical trials website^c^ and PND
	R.Test Evolution 4		Novacor	AD	>1 month	2	Yes	Clinical trials website and PND
	Cardio ID+ Digital Holter Recorder 153+ Series		Rozinn	M+R	>1 day	3	Yes	PND
	SenseLink^a^		Temeco	AD+ER	>1 week	5	Yes	—
	Zensor		Renew Health Ltd	AD	>1 week	7	Yes	Clinical trials website^c^ and PND
	1AX Wearable Biosensor^a^		LifeSignals Inc	M+R	>1 day	6	No	Clinical trials website^c^ and PND
	ATPatch		Atsens	AD	>1 week	3	No	Clinical trials website and PND
	BodyGuardian		Preventice Technologies Inc	AD	1 day	4	No	Clinical trials website and PND
	Cardea Solo^a^		Cardiac Insight Inc	AD	1 week	2	Yes	Clinical trials website^c^ and PND
	Cardio STAT		Icentia Inc	AD	>1 week	2	Yes	Clinical trials website
	Nuvant Mobile Cardiac Telemetry Monitor		Corventis	AD+CD^k^	>1 week	—	—	Clinical trials website^c^ and PND
	S-Patch Ex^a^		Wellysis	M+R	>1 day	2	No	Clinical trials website^c^
	Tag ECG^a^		Welch Allyn	AD	1 week	2	Yes	—
	Trident Holter Patch^a^		TZ Medical Inc	M+R^g^	1 week	—	Yes	—
	Zio XT		iRhythm Technologies Inc	M+R	>1 week	2	Yes	Clinical trials website and PND
	CardioMem CM 3000-12BT^a^		GE Healthcare	AD	>1 day	12	Yes	Clinical trials website and PND
	MCOT^a^		Koninklijke Philips NV	AD	>1 day	4	No	Clinical trials website^c^
	BardyDX CAM^a^		Bardy Diagnostics Inc	AD	>1 week	2	Yes	PND
**Wearable**								
	QardioCore^a^		Qardio Inc	M+R	1 day	4	No	PND
	CART-I^a^		Sky Labs Inc	AFD^l^	1 day	—	No	Clinical trials website^c^
	Hexoskin		Carré Technologies Inc	Research^g^	>1 day	—	No	Clinical trials website^c^
	VitalJacket Holter		BioDevices SA	AD+CD	>1 day	6	Yes	—
	X10X and X10Y		L.I.F.E. Italia Srl	—	1 day	8	Yes	Clinical trials website^c^
	Master Caution		HealthWatch Ltd	M+R	—	12	No	Clinical trials website^c^ and PND
	Zephyr		Medtronic	M+R	>1 day	2	Yes	PND

^a^Device found via internet search.

^b^M+R: measure and record electrocardiogram.

^c^No results available.

^d^PMD: pacemaker detection.

^e^Not available.

^f^HRD: heart rate detection.

^g^Data found via internet search.

^h^PND: Food and Drug Administration 510(k) Premarket Notification database.

^i^ER: event recorder.

^j^AD: arrhythmia detection.

^k^CD: conduction disorder detection.

^l^AFD: atrial fibrillation detection.

**Table 2:** Functionality characteristics per device (intermittent recording devices).

Device			Manufacturer	Intended use			Recording time		Number of electrodes			Stand-alone			Source of clinical evaluation evidence	
**Handheld**																
	Afib Alert	Lohman Technologies LLC		AFD^a^			<1 minute		2		Yes				PND^b^	
	Beurer ME 80	Beurer GmbH		AD^c^			<1 minute		1		Yes				—^d^	
	Cardea 3^e^	Human Medical Solutions Inc		AD			<1 minute		4		No				—	
	Cardio-B Palm ECG^e^	Shanghai International Holding Corp		M+R^f^			<1 minute		2		Yes				—	
	Coala Heart Monitor	Coala Life AB		AFD			<1 minute		3		No				Clinical trials website and PND	
	ECG Check^e^	Cardiac Designs Inc		AD			<1 minute		2			No			PND	
	Eko DUO^e^	Eko Devices Inc		M+R			>1 minute		2			No			Clinical trials website and PND	
	Impulse ECG	—		—			<1 minute		2			No			—	
	Istel HR-2000^e^	Diagnosis SA		M+R			<1 minute		4			No			Clinical trials website	
	KardiaMobile	AliveCor, Inc		AD			<1 minute		2			No			Clinical trials website and PND	
	KardiaMobile 6L	AliveCor, Inc		AD			<1 minute		3			No			Clinical trials website and PND	
	KardiaMobile Card	AliveCor, Inc		AD			<1 minute		2		No				Clinical trials website and PND	
	MyDiagnostick	MyDiagnostick Medical BV		AFD			1 minute		2		Yes				Clinical trials website	
	HeartCheck PEN	CardioComm Solutions, Inc		AD			<1 minute		2		No				PND	
	Zenicor-ECG	Zenicor Medical Systems		M+R			<1 minute		2		No				Clinical trials website	
	Heartscan HCG-801^e^	Omron		M+R			<1 minute		3			Yes			Clinical trials website	
**Patch**																
	VitalPatch RTM^d^	VitalConnect Inc		M+R			<1 week		2		Yes				PND	
**Wearable**																
	Nuubo ECG Vest	Nuubo Wearable Technologies			M+R		>1 week		4				Yes		Clinical trials website	
	SmartHeart^e^	SHL Telemedicine International Ltd		M+R			<1 minute		18			No			PND	
	Apple Watch	Apple Inc		AFD			<1 minute		2			Yes			Clinical trials website and DND^g^	
	Move ECG^e^	Withings		AFD			<1 minute		2			No			Clinical trials website and PND	
	Galaxy Active Watch (2 and 3)^e^	Samsung Electronics Co, Ltd				AFD		<1 minute		2				Yes		Clinical trials website and PND
	Venu 2 Plus Smartwatch^e^	Garmin Ltd				AFD		<1 minute		2				Yes		PND
	Fitbit Charge 5 ^e^	Alphabet Inc				AFD		<1 minute		2				Yes		Clinical trials website and PND

^a^AFD: atrial fibrillation detection.

^b^PND: Food and Drug Administration 510(k) Premarket Notification database.

^c^AD: arrhythmia detection.

^d^Not available.

^e^Device found via internet search.

^f^M+R: measure and record electrocardiogram.

^g^DND: Food and Drug Administration 510(k) De Novo database.

### Use Characteristics

Adhesive patch devices are intended to be placed either at the left side (11/28, 39%) or center of the chest (17/28, 60%). Table 2 shows that these devices require setup for positioning the devices on patients, with the steps including skin preparation (shaving and removal of nonconductive skin layer via skin abrasion) as well as templates for the correct device placement, and for performing successful patient recordings. These steps are performed once because these devices are used on a longer-term basis (from >1 day up to >30 days). There are 2 types of patch devices: those in which the whole system, including 2 or 3 electrodes, is embedded in the patch and those that use disposable single electrodes attached through a cable. In the latter case, the devices aim to provide recordings that resemble 12-lead clinical ECG recordings. Patch devices are usually managed by health care centers, and analyses are performed by the manufacturer, specialized companies, or at health care centers.

The wearables category has shown to be more versatile because some of the devices (7/14, 50%) in this category are intended for intermittent use, whereas others (7/14, 50%) are intended for continuous recording. Devices in the former category are often used as daily accessories, such as the Apple Watch (Apple Inc), Amazfit Band (Zepp Health Corporation), and CART-I smart ring (Sky Labs Inc). As for the wearable devices that offer continuous recording, they can be used as garments such as T-shirts. These devices have embedded textile electrodes and can perform recordings lasting 24 hours. Although these devices offer prolonged recordings compared with the accessory wearables, only 2 (29%) of the 7 devices allow simultaneous recording and analysis.

Handheld devices are designed for patients, both for clinical and home use. Of the 14 devices, 11 (79%) rely on limb (including lower limbs) recordings, whereas 3 (21%) perform chest recordings. To record ECGs using handheld devices, no extra steps are required for preparing the area of contact, and ECGs can be recorded in <1 minute.

### Technical Characteristics

Patch and wearable devices can be used for at least 24 hours continuously, and these devices can include the feature to detect cardiac events automatically. By contrast, handheld devices have recording durations, initiated by patients, ranging from 15 to 120 seconds. Patients are typically instructed to perform recordings at the onset of symptoms or at specified times.

Handheld devices record ECGs via dry electrodes. These metal electrodes are manufactured from stainless steel, copper, silver or silver chloride (Ag or AgCl), or other unspecified materials. By contrast, patch devices use disposable electrodes, either commercially available or as part of the product.

Of the 58 devices with available manufacturer information, 43 (74%) are intended to be used at home. Of these 43 devices, only 21 (49%) disclosed their IP rating. Of these 21 devices, 5 (24%) have been tested for IP22 (protected from touch by fingers and objects >12 mm and protected from water spray <15° from the vertical) and 10 (48%) for higher IP, whereas 6 (29%) devices have been tested only for water IP (Tables 3 and 4).

**Table 3:** Technical characteristics per device (continuous recording devices).

Device		Use environment	Multiple measures	Requires setup	Ingress protection rating	Sampling rate (Hz)	Resolution (bits)	Signal bandwidth (Hz)
**Patch**								
	C3 Holter Monitor	Home and clinical	N/A^a^	✓	—^b^	256	24	—
	Custo Guard Holter	Clinical	✓	✓	65	533	18	0-105
	D-Heart	Home and clinical	N/A	✓	—	640	—	—
	DigiTrak XT	Home	N/A	✓	—	175	10	0.05-60
	DR400 Patch Holter	Home	✓	✓	44	180	12	0.05-70
	Beam ECG Mobil	Home	✓	✓	—	200	12	Event: 0.3-75; loop: 0.1-75
	Bittium Faros 180	Home	✓	✓	67	100	—	—
	H3+ Digital Holter	Clinical	N/A	✓	—	180	12	—
	M-trace Mini ECG	Home	N/A	✓	—	1000	24	0.5-100
	PocketECG	—	N/A	✓	22	300	—	0.05-60
	R.Test Evolution 4	—	N/A	✓	X4	200	10	—
	Cardio ID+ Digital Holter Recorder 153+ Series	—	N/A	N/A	—	1024	12	0.05-60
	SenseLink	Home and clinical	N/A	✓	22	1000	16	—
	Zensor	—	N/A	✓	22	360	12	0.67-40
	1AX Wearable Biosensor	Home	N/A	✓	24	244.14	16	0.2-40
	ATPatch	Home	N/A	✓	57	250	10	0.05-40
	BodyGuardian	Home	✓	✓	X4	256	12	—
	Cardea Solo	Home	N/A	✓	—	—	—	—
	Cardio STAT	Home	N/A	N/A	—	—	—	—
	Nuvant Mobile Cardiac Telemetry Monitor	—	N/A	✓	—	200	10	—
	S-Patch Ex	—	N/A	✓	55	256	12	—
	Tag ECG	Home	✓	✓	X7	250	—	0.05-65
	Trident Holter Patch	—	N/A	N/A	—	—	—	—
	Zio XT	—	✓	✓	X4	200	10	0.05-30
	CardioMem CM 3000-12 BT	Home	N/A	✓	20	1024	12	0.05-120
	MCOT	Home	N/A	✓	X4	250	12	—
	BardyDX CAM	Home	N/A	✓	23	171	—	0.67-25
**Wearable**								
	QardioCore	Home	N/A	N/A	65	600	16	0.05-40
	CART-I	Home	N/A	N/A	58	—	—	—
	Hexoskin	Home	N/A	N/A	—	256	12	—
	VitalJacket Holter	—	N/A	✓	—	500	10	0.03-150
	X10X and X10Y	—	N/A	N/A	—	—	—	—
	Master Caution	Home	N/A	N/A	—	1000	—	—
	Zephyr	Home	N/A	N/A	55	250	12	—

^a^N/A: not applicable.

^b^Not available.

**Table 4:** Technical characteristics per device (intermittent recording devices).

Device			Use environment		Multiple measures		Requires setup		Ingress protection rating		Sampling rate (Hz)		Resolution (bits)		Signal bandwidth (Hz)
**Handheld**															
	Afib Alert	Home		✓		✓		—^a^		—		—		—	
	Beurer ME 80	Home		✓		N/A^b^		—		256		—		—	
	Cardea 3	Home and clinical		N/A		N/A		—		500		—		1-75	
	Cardio-B Palm ECG	Home		✓		N/A		—		—		—		1-40	
	Coala Heart Monitor	Home and clinical		✓		N/A		22		1000		24		—	
	ECG Check	Home		N/A		N/A		—		200		—		0.5-25	
	Eko DUO	Clinical		N/A		N/A		55		—		—		—	
	Impulse ECG	Home		N/A		N/A		—		—		—		—	
	Istel HR-2000	Home		N/A		N/A		22		160, 320, and 640		24		0.05-32, 0.05-35, and 0.05-130	
	KardiaMobile	Home		N/A		N/A		64		300		16		0.5-40	
	KardiaMobile 6L	Home		✓		N/A		22		300		16		0.5-40	
	KardiaMobile Card	Home		N/A		N/A		X8		300		16		0.5-40	
	MyDiagnostick	Clinical		N/A		N/A		24		—		—		—	
	HeartCheck PEN	Home		✓		N/A		—		250		—		1-40	
	Zenicor-ECG	Home		N/A		N/A		22		—		—		—	
	Heartscan HCG-801	Home		✓		N/A		20		125		—		0.05-40	
**Patch**															
	VitalPatch RTM	Home and clinical		✓		✓		24		—		—		—	
**Wearable**															
	Nuubo ECG Vest	Home and clinical		N/A		N/A		22		250		—		0-65	
	SmartHeart	Home		N/A		✓		—		—		—		0.05-150	
	Apple Watch	Home		N/A		N/A		—		—		—		—	
	Move ECG	Home		N/A		N/A		—		—		—		—	
	Galaxy Active Watch (2 and 3)	Home		N/A		N/A		—		—		—		—	
	Venu 2 Plus Smartwatch	Home		N/A		N/A		—		—		—		—	
	Fitbit Charge 5	Home		N/A		N/A		—		—		—		—	

^a^Not available.

^b^N/A: not applicable.

### Clinical Evidence

Through the individual searches with regard to all 58 devices for which we were able to find characteristics from manufacturer websites, we found articles (n=36) that covered 22 (38%) of the devices, demonstrating their capabilities to record cardiac disorders. Of these 22 devices, 8 (36%) are handheld devices, 8 (36%) are patch devices, and 6 (28%) are wearables (Tables 5 and 6).

**Table 5:** Summary of device study objectives (continuous recording devices).

Device, study, heart disorder or ECG^a^ abnormality					Comparators		Participants, n		Sensitivity, %		Specificity, %		Accuracy, %
**Patch**													
	**D-Heart**												
		**Maurizi et al [14]**											
			Quality recordings	12-lead device		117		—^b^		—		—	
	**Bittium Faros 180**												
		**Müller et al [15]**											
			AF^c^	PPG^d^ devices		144		90		84.2		—	
	**R.Test Evolution 4**												
		**Eysenck et al [16]**											
			AF	Zio XT, Nuubo ECG Vest, and BardyDX CAM		21		—		—		30-second recording: 50.8; 6-minute recording: 77.3	
	**CardioSTAT**												
		**Nault et al [17]**											
			AF	12-lead Holter		212		—		—		99	
	**Zio XT**												
		**Eysenck et al** **[16]**											
			AF	Zio XT, Nuubo ECG Vest, BardyDX CAM, and R.Test Evolution 4		21		—		—		30-second recording: 86.7; 6-minute recording: 80.8	
		**Hannun et al [18]**											
			AF and flutter	—		53,549		71		94.1		—	
			AVB^e^	—		53,549		73.1		98.1		—	
			Bigeminy	—		53,549		82.9		99.6		—	
			EAR^f^	—		53,549		38		99.3		—	
			IVR^g^	—		53,549		61.1		99.1		—	
			Junctional rhythm	—		53,549		63.4		98.4		—	
			Noise	—		53,549		74.9		98.3		—	
			Sinus rhythm	—		53,549		90.1		85.9		—	
			SVT^h^	—		53,549		40.8		98.3		—	
			Ventricular tachycardia	—		53,549		65.2		99.6		—	
			Wenckebach	—		53,549		54.1		98.6		—	
	**BardyDX CAM**												
		**Eysenck et al** **[16]**											
			AF	Zio XT, Nuubo ECG Vest, BardyDX CAM, and R.Test Evolution 4		21		—		—		30-second recording: 99.9; 6-minute recording: 95.3	
	**ATPatch**												
		**Choi et al [19]**											
			Quality recordings	12-lead device		10		—		—		0.1	
	**BodyGuardian**												
		**Bruce et al [20]**											
			Quality recordings	—		10		97		77		—	
**Wearable**													
	**Amazfit**												
		**Chen et al [21]**											
			AF	PPG devices		451		87.3		99.2		94.76	
		**Zhang et al [22]**											
			Rhythm disorders and AF	12-lead device		291		93.3		95.3		—	
			PAC^i^	12-lead device		291		84		96.6		—	
			PVC^j^	12-lead device		291		89.3		93.9		—	
			First-degree AVB	12-lead device		291		32.1		97.7		—	

^a^ECG: electrocardiogram.

^b^Not available.

^c^AF: atrial fibrillation.

^d^PPG: photoplethysmography.

^e^AVB: atrioventricular block.

^f^EAR: ectopic atrial rhythm.

^g^IVR: idioventricular rhythm.

^h^SVT: supraventricular tachycardia.

^i^PAC: premature atrial contraction.

^j^PVC: premature ventricular contraction.

**Table 6:** Summary of device study objectives (intermittent recording devices).

Device, study, heart disorder or ECG^a^ abnormality					Comparators		Participants, n		Sensitivity, %		Specificity, %		Accuracy, %
**Handheld**													
	**Beurer ME 80**												
		**Nigolian et al [23]**											
			AF^b^	12-lead device		16		100		94		96	
			AVB^c^	12-lead device		13		85		97		94	
			LBBB^d^	12-lead device		7		71		100		96	
			RBBB^e^	12-lead device		10		90		100		98	
			LVH^f^	12-lead device		5		80		100		98	
			ST-segment elevation	12-lead device		11		64		93		87	
			ST-segment depression	12-lead device		13		54		95		85	
			Prolonged QTc	12-lead device		4		50		91		88	
	**Coala Heart Monitor**												
		**Insulander et al [24]**											
			AF	—^g^		1000		95.1		97.6		97.3	
	**ECG Check**												
		**Aljuaid et al [25]**											
			AF	Holter		90		100		97		—	
	**Eko DUO**												
		**Bokma et al [26]**											
			CHD^h^	12-lead device, Move ECG, KardiaMobile		176		100		99		—	
		**Bachtiger et al [27]**											
			LVEF^i^ of ≤40%	12-lead device		1050		91.9		80.2		—	
	**Istel HR-2000**												
		**Krzowski et al [28]**											
			Sinus rhythm	12-lead device and KardiaMobile		98		91.5		84.6		—	
			AF	12-lead device and KardiaMobile		98		77.3		98.7		—	
	**KardiaMobile**												
		**Krzowski et al [28]**											
			Sinus rhythm	12-lead device and Istel HR-2000		98		88.1		89.7		—	
			AF	12-lead device and Istel HR-2000		98		86.4		97.4		—	
		**Palà et al [29]**											
			AF	WatchBP, MyDiagnostick, and FibriCheck		359		80		95.5		—	
		**Lau et al [30]**											
			AF	12-lead device		109		97.5		92		94.5	
		**Desteghe et al [31]**											
			AF (cardiology ward)	12-lead device and MyDiagnostick		445		54.5		97.5		—	
			AF (geriatric ward)	12-lead device and MyDiagnostick		445		78.9		97.9		—	
		**Bokma et al [26]**											
			CHD	12-lead device, Move ECG, and Eko DUO		176		100		99		—	
		**Scholten et al [32]**											
			AF	12-lead device, Apple Watch, and Move ECG		220		99		97		—	
		**Bumgarner et al [33]**											
			AF	12-lead device		100		99		83		—	
		**Ford et al [34]**											
			AF	Apple Watch		125		94		90		91	
		**Wasserlauf et al [35]**											
			AF	Implantable Cardiac Monitor		24		83.3		83.3		—	
		**Himmelreich et al [36]**											
			AF and AFL^j^	12-lead device		23		100		100		—	
			AF	12-lead device		44		90.9		93.5		—	
			AF	12-lead device		28		46.4		100		—	
	**MyDiagnostick**												
		**Tieleman et al [37]**											
			AF	12-lead device		192		100		95.9		—	
		**Palà et al [29]**											
			AF	WatchBP, KardiaMobile, and FibriCheck		359		76.9		97.1		—	
		**Verbiest-van Gurp et al [38]**											
			AF	12-lead device and WatchBP		4339		90.1		97.9		—	
		**Vaes et al [39]**											
			AF	12-lead device		191		94		93		—	
		**Yeo et al [40]**											
			AF	12-lead device		671		100		96.2		—	
		**Karregat et al [41]**											
			Paroxysmal AF	12-lead Holter		270		66.7		68.8		—	
		**Desteghe et al [31]**											
			AF (cardiology ward)	12-lead device and KardiaMobile		445		81.8		94.2		—	
			AF (geriatric ward)	12-lead device and KardiaMobile		445		89.5		95.7		—	
	**Zenicor-ECG**												
		**Doliwa et al [42]**											
			AF	12-lead device		49		96		92		—	
**Wearable**													
	**Apple Watch**												
		**Abu-Alrub et al [43]**											
			AF	Galaxy Watch Active 3, and Move ECG		100		87		86		—	
		**Nasarre et al [44]**											
			Cardiac abnormalities and cardiac arrest	12-lead device		67		100		100		—	
			Brugada Syndrome	12-lead device		67		92		100		—	
			Long QT	12-lead device		67		80		100		—	
			HCM^k^	12-lead device		67		92		85		—	
			ARVC/D^l^	12-lead device		67		100		99		—	
		**Caillol et al [45]**											
			Bradyarrhythmias	12-lead device		40		96		91		—	
			Tachyarrhythmias	12-lead device		40		25		99		—	
			Cardiac Ischemia	12-lead device		40		7		100		—	
		**Spaccarotella et al [46]**											
			Measurements of the QT interval	12-lead device		—		69		88		—	
		**Scholten et al [32]**											
			AF	12-lead device, Move ECG, and KardiaMobile		220		96		94		—	
		**Ford et al [34]**											
			AF	KardiaMobile		125		68		93		87	
	**Move ECG**												
		**Bokma et al [26]**											
			CHD	12-lead device, KardiaMobile, and Eko DUO		176		100		98		—	
		**Abu-Alrub et al [43]**											
			AF	Apple Watch, Galaxy Watch Active 3		100		88		81		—	
		**Scholten et al [32]**											
			AF	12-lead device, Apple Watch, and KardiaMobile		220		95		95		—	
	**Nuubo ECG Vest**												
		**Eysenck et al [16]**											
			AF	Zio XT, Nuubo ECG Vest, BardyDX CAM, and R.Test Evolution 4		21		—		—		30-second recording: 97; 6-minute recording: 89.7	
	**Fitbit**												
		**Rajagopalan [47]**											
			AF	12-lead device		475		98.7		100		—	
	**Galaxy Active Watch**												
		**Yang [48]**											
			AF	12-lead device		544		98.1		100		—	

^a^ECG: electrocardiogram.

^b^AF: atrial fibrillation.

^c^AVB: atrioventricular block.

^d^LBBB: left bundle branch block.

^e^RBBB: right bundle branch block.

^f^LVH: left ventricular hypertrophy.

^g^Not available.

^h^CHD: congenital heart defect.

^i^LVEF: low ventricular ejection fraction.

^j^AFL: atrial flutter.

^k^HCM: hypertrophic cardiomyopathy.

^l^ARVC/D: arrhythmogenic right ventricular cardiomyopathy/dysplasia.

Of the 36 articles, 24 (66%) evaluated the devices’ capabilities to diagnose rhythm disorders [15-17,21,24,25,29-35,​^37^-^43^,^49^-^56^], whereas 7 (20%) reported on capabilities to detect rhythm disorders and other heart conditions, such as cardiomyopathy, conduction disorders, and cardiac ischemia [18,22,23,28,36,45,57]. Finally, for 3 (14%) of the 22 devices, the evidence found was regarding the evaluation of the quality of the signals recorded by them [14,19,20].

In 23 (63%) of the 36 articles, the feasibility to detect AF was studied. Studies on the Istel HR-2000 (Diagnosis SA), AliveCor heart monitor (AliveCor Inc), MyDiagnostick (MyDiagnostick Medical BV), Apple Watch (Apple Inc), Amazfit (Zepp Health Corporation), and Move ECG (Withings France SA) have reported sensitivities of <94% (range 54.5%-94%) for the detection of AF [21,28,29,31,34,43]. The Apple Watch, KardiaMobile (AliveCor), Bittium Faros 180 (Bittium), and Move ECG studies reported specificities of <90% (range 81%-86%) for AF detection [15,33,43]. For other rhythm disorders, the studies reported specificities of >85% (range 85.9%-99,6%), whereas the sensitivities ranged from 25% to 96% [18,22,45]. For cardiac ischemia detection, the Apple Watch and Beurer ME 80 (Beurer GmbH) were evaluated, and the studies reported specificities of >90% (range 93%-100%) and sensitivities of <65% (range 7%-64%); these studies included 40 and 13 participants, respectively [23,45].

## Discussion

### Overview

The aim of this review was to provide an overview of the mobile ECG devices available in the market, including the technology used, their clinical application, and the published clinical evidence. In this review, we have identified 58 mobile ECG devices with available manufacturer information and observed that the main intended use of these devices is the detection of rhythm disorders, more specifically AF. We analyzed the relation of the technical characteristics and how these design decisions influence the capabilities of the devices to record cardiac disorders. In terms of clinical evidence, upon reviewing 2 FDA databases, we noted that most of the devices (33/58, 57%) did not require clinical validation because they have been found to be equivalent to other ECG devices in the market or to their previous versions. The published studies we found focused on the evaluation of the devices for the detection of rhythm disorders, more specifically AF.

### Clinical Purpose and Technical Capabilities of ECG Devices

To detect rhythm disorders, especially AF, one may only need to be able to capture basic heart rhythms. However, according to the European Society of Cardiology guidelines for the diagnosis of AF, an irregular R-R interval, absence of distinct repeating P waves, and irregular atrial activations indicate AF [58]. For home monitoring devices, it is not completely clear whether the detection of AF is solely based on the detection of irregular R-R intervals or whether they include P-wave detection as well.

For the diagnosis of AF, a 12-lead ECG is recorded only if the physician suspects AF, and the diagnosis will be provided if the ECG records an AF episode [59]. With our analysis, it is also possible to note that continuous monitoring devices show better performance in terms of sensitivity and specificity than intermittent monitoring devices. By using continuous recording systems, the chances of recording AF events are high, but it is necessary to consider that the amounts of data generated while continuous recordings could make it cumbersome to see when the AF events have been detected, as recordings are performed over periods higher than 24 hours.

For wearable devices with continuous recording systems, 16% of the recordings have been considered inconclusive by cardiologists, according to third-party comparisons [60]. It has also been reported that owing to signal processing and algorithm settings, devices such as the Apple Watch are limited in terms of diagnosing and misdiagnosing AF in comparison with medical grade devices [61]. This brings into question the value of using wearable devices with continuous recording systems for early detection of AF because these devices add more challenges to already stressed health care systems and clinical workflows [12].

Considering the clinical importance of the detection of AF as well as cardiac ischemia, the difference in the number of devices that can detect either condition is striking. The overwhelming number of devices is aimed at detecting AF, whereas no devices are intended for the detection of ischemia, and only 2 (9%) of the 22 devices have published studies for the detection of ST-segment elevation. From a technical point of view, many of the devices may not be completely limited in their capability to detect ischemia.

To detect other cardiac disorders such as ischemia, one also needs to be able to capture morphological details of the ECG. For either case, there are some technical challenges that require further discussion and are discussed in the following paragraphs.

A low number of electrodes and limited measurement area impose restrictions on the detection of all heart diseases [45,62,63]. Caillol et al [45] have shown how single-lead devices such as the Apple Watch could miss ST-segment elevation caused by ischemia in specific parts of the heart. The authors were able to demonstrate that ST-segment elevations and depressions were visible for lateral and inferior infarction, but when they attempted to record an anterior infarction, no ST-segment elevations or depressions were visible on the recordings [45]. Samol et al [64] demonstrated that performing ECG recordings with the Apple Watch (placed on the chest) allowed 6 precordial channels to be recorded in a serial manner [64]. A study using the AliveCor heart monitor showed that with only 2 electrodes, the device is capable of recording ST-segment elevations, once again by performing serial recordings [65,66]. The other device studied for ischemia detection (Beurer ME 80) also requires serial measurements. Considering the acute nature of the condition, we do not see any feasible application of this method of measuring ECGs for ischemia detection.

There is a need for ECG devices intended to detect ischemic diseases, but, as our search has shown, there are no devices intended for this purpose available in the market. There is evidence showing that methods for measurements and technologies are moving toward the detection of ischemic diseases; for example, the RELF method (in which the RELF leads record the voltage differences from the right shoulder [R] to an exploratory electrode [E], to the left shoulder [L], and to the left iliac crest [F]) has been developed using a 3-lead detection system, and when this device was tested for the detection of acute coronary artery occlusion, it showed a specificity of 96% during daily life recordings, and when ST-segment elevation myocardial infarction (STEMI) criteria on a 12-lead ECG device were observed during the interventions, the RELF method had a sensitivity of 100% [67,68].

For heart diseases other than rhythm disorders, single-lead devices allow preliminary recordings to be made, but to obtain a deeper understanding and to allow physicians to provide a diagnosis, more information should be recorded. However, one can imagine that performing studies on conditions such as myocardial infarction in acute settings, is more complex. Furthermore, the approval of a device aiming to detect a high-risk cardiac disorder would require compliance with more stringent requirements; for example, upon the detection of heart disorders such as acute coronary syndrome, it is necessary to provide rapid attention and therapy for patients. If such disorders are undetected, the life and quality of life of patients will be highly affected.

Other characteristics such as signal processing and acquisition may affect the capabilities of the device to detect various cardiac disorders. Applying filters to the captured ECG affects the waveform, which could lead to misinterpretation and misdiagnosis. Signal processing is a design characteristic that has been specified in the International Electrotechnical Commission (IEC) standard [69]. For the detection and interpretation of ischemic diseases, devices need to be able to record changes in the ST segment. The suggested update on the current standard for ECG devices specifies that devices that contain a filter with a high-pass cutoff frequency of 0.67 Hz can detect ST-segment deviations as long as filters are not modifying the phase of the ECG signal [70]. Bailey et al [71] have performed measurements to demonstrate that zero-phase filters indeed do not modify the phase of the ECG recordings. For 45% (26/58) of the devices that specify signal bandwidth, the lower limit ranges from 0.03 to 1 Hz, whereas the upper limit ranges from 25 to 1000 Hz. According to the suggested update of the standard, 17 (65%) of the 26 devices would be suitable for ischemia detection. In addition, the applied filter (zero phase or not) will influence the ability to detect ischemia; however, this is not specified for any of the devices.

Regarding signal acquisition, one of the influencing factors is the sampling frequency, which refers to the time interval of the discrete digital points transformed from the cardiac biopotentials [72]. In general, mobile ECG devices have a sample frequency of at least 250 samples per second. The applicable standard for home use does not specify the required sample frequencies [73]. The general standard for ECG devices used in the clinical environment recommends sample frequencies of at least 500 samples per second (there is no specification for devices used at home) [69,74]. According to Kligfield et al [72], most of the diagnostic information in the ECG is contained below 100 Hz in adults. We noted that all devices analyzed are capable of recording cardiac biopotentials at adequate sample rates.

Besides sampling frequency, signal resolution influences the quality of the ECG. The signal resolution refers to how biopotential signals are expressed in digits into which the input signal can be converted, based on the number of discrete steps. When a device has a resolution of 16 bits, it means that the number of measured steps between the minimum and maximum values that can be recorded is 2^16^=65,536. In other words, if a device can only capture signals between −2.5 V and +2.5 V (5 V at full-scale deflection), the detail that can be recorded at a resolution of 24 bits is 0.298 µV, also referred to as the least significant bit (LSB). Thus, the combination of full-scale deflection and resolution determine how little of the heart biopotentials can be captured. In fact, the question is this: what is the maximum value of the LSB that provides enough detail on the morphology of the ECG signals (the standard defines an LSB of ≤1 µV [69])?

The relationship of sampling frequency and signal resolution is relevant for diagnosis because of the added information that these features provide to physicians; for example, regarding the relationship of fragmented QRS (fQRS) and heart disorders, fQRS can only be observed when the sample rate and resolution are sufficient to capture the detailed signals. It has been demonstrated that the use of fQRS is a key feature for detecting myocardial scars in patients [75-77].

### Device Features and Technical Characteristics

It is observed that the prioritization of a device’s characteristics depends upon its intended use. For handheld home-use devices, usability and easy-of-use characteristics are a priority in comparison with patches, where the recording is a priority and the comfort of the patient is secondary.

We believe that the prioritization regarding users starts from design decisions, such as the selection of electrodes. Adhesive patch devices use wet electrodes, whereas handheld devices and wearables use a mix of electrodes, ranging from embedded metal dry electrodes to textile electrodes that do not require skin preparation. According to electrode comparisons and reviews, wet gel electrodes provide good signal quality for short-term recordings because the gel improves the electrode-skin contact, allowing the formation of a conductive path between skin and electrode [78,79]. However, it has also been noted that long-term use of these types of electrodes can cause skin irritation, and the signal quality decreases as the conductive gels dry out [80]. Hickey et al [81] have reported that by using devices that include multiple adhesive electrodes or patch-type devices, user compliance is diminished owing to application and wearing complexity. Dry electrodes do not require a medium for conduction because the substrate is in direct contact with the skin. This metal-skin interface has been reported to influence the quality of recorded signals owing to movement artifact and charge sensitivity [79]. When biocompatible, the use of dry electrodes prevents undesirable chemical effects and skin irritation on patients [78,82].

For home-use medical electrical devices, their enclosures should provide the user protection against access of hazardous parts inside the enclosure and against harmful effects owing to ingress of water [83]. To designate a device’s degree of protection, the IP rating is disclosed. Devices must comply with the minimum IP rating of 22, which is applicable to medical home-use and health care devices [73]. Compliance with the features specified in the standard helps to guarantee the essential device performance as well as basic device safety to users and patients. Of the 38 devices meant for home use, only 14 (37%) have disclosed their IP rating in compliance with the applicable IEC standard. For the remaining devices (24/38, 63%), the IP rating has not been registered on the available device patient information; however, this requirement might be covered by the checklist of general safety and performance requirements. Of note, the devices carry the Conformité Européenne (CE) marking and meet the requirements specified by IEC standards [73,84].

### Clinical Evidence

Of the 58 devices with available manufacturer information, only 18 (31%) have published feasibility and reliability studies on diagnosing heart conditions. Patch devices are used as the benchmark for comparison in clinical studies, specifically if these devices have a continuous recording function (Holter devices). As for other devices with published clinical evidence, these devices perform recordings in positions that are not similar to those of the 12-lead clinical ECG. The studies are part of the clinical evidence on the route to compliance with medical device regulations for clinical testing to show the capability of the device to achieve its intended purpose, clinical performance, and benefits [85]. Upon performing the search in the FDA 510(k) Premarket Notification database, we noted that most devices do not include clinical evidence in the submissions because they show evidence of their similarities to other devices in the market or previous versions of the device in question. However, for recently released devices, such as the Apple Watch, we were able to see detailed clinical evidence summaries. We believe that because compliance requirements for new devices have become more stringent, we can expect to see more clinical evidence for new devices. Our belief is also based on the changes made to the European Union regulations governing medical devices; as other researchers have pointed out, the new regulations focus on the need for more clinical data for all medical devices [86,87].

In 2017, the medical device directive was updated to a new version, which is more stringent and aims to improve the safety and effectiveness of medical devices. One of the main changes made to the regulations concerns the additional emphasis placed on the clinical evidence of medical devices [83] to ensure their safety and effectiveness [85]. For devices without available clinical evidence, we could argue that for them to be available in the market, an important step is the clinical evaluation. Before 2017, owing to their similarities to other products already available in the market, these devices’ clinical investigations could have been based on the clinical evidence presented by similar devices. This is specifically the case if these devices have been certified before May 2021, when the European Union Medical Device Regulation became fully applicable. Nowadays, another source of information regarding the performance and safety of medical devices as well as the risks involved in using them is the postmarket surveillance; however, these activities are normally confidential, which could be the reason for the lack of available public clinical data for these devices [88].

The studies have shown promising evidence of the capabilities of the ECG devices, but they have been tested on small populations, which is a limitation in terms of investigating their full functionality and use in broader scenarios. As has been specified in the guidance regarding sufficient clinical evidence, these types of publications could be sufficient if there are no concerns regarding the safety of the patient and performance of the device [88].

For handheld devices, another observation concerned the design of the studies owing to the use characteristics of these devices. In the study by Magnusson et al [89], the recordings were limited and scheduled at certain times of the day, limiting the comparison with patch devices, which were used on a continuous basis, whereas in another study, the approach was based on patient management, with patients instructed to perform recordings when symptoms were present, which, as Doliwa et al [90] have shown, is an improvement with regard to detecting paroxysmal AF in patients who have had a recent stroke. These data were confirmed by other studies with similar approaches and outcomes [41,91]. The design of clinical studies should take into account user case scenarios that approximate to the intended use of the device in daily life. By designing studies based on user scenarios, it would be possible to compare the capabilities of handheld devices with those of patch devices when their performance is evaluated for the detection of symptomatic cardiac diseases. There is a marked lack of studies for the vast majority of the devices (35/58, 60%) included in this review, with, as mentioned previously, only 38% (22/58) of the devices having been investigated in studies regarding their capabilities to detect cardiac disorders.

### Limitations

To the best of our ability, we tried to perform an exhaustive search to identify all available devices; however, we cannot guarantee that all were indeed identified. In terms of the analysis performed, we were not able to summarize all technical and clinical information related to the devices owing to the lack of availability of data for such devices.

As this review shows, there is a wide range of mobile ECG devices available for home use, but as mentioned, technologies are moving toward the use of other sensors. One limitation of this review is that we have not analyzed other devices containing other types of sensors used for cardiac monitoring, such as photoplethysmography or consumer electronics not intended for detection of cardiac disorders such as the Fitbit (Google LLC); however, this was a choice because we decided to include only ECG devices.

Finally, in our analysis, we decided not to include the fact that for some devices (ie, KardiaMobile and Fitbit), users are required to sign up for subscription services to obtain further diagnosis of ECGs. We decided not to analyze the availability of these types of services because they depend on location, and prices may change over time.

### Future Perspectives

We believe that the inclusion of other sensors will help to improve ECG devices’ capabilities to detect disorders. As noted by Sana et al [92], certain heart conditions are difficult to detect with ECG recordings [92]. Structural heart abnormalities can potentially be diagnosed with the help of other sensors (eg, by analyzing sound, accelerometer, and gyroscope recordings), which suggests that phonocardiograms or seismocardiograms could be added to the ECG recording [92]. We noted that, of the 58 devices included in this review, a few (n=2, 3%), such as the Eko DUO (Eko Devices Inc) and Coala Heart Monitor (Coala Life AB), already include these features. We also noted that there is a trend toward acquiring more information on the heart. During the systematic search, we came across prototypes, which included microphones, accelerometers, and gyroscope sensors, that are currently in development and in early stages of testing [93-97].

### Conclusions

In this review, we have explored the current scope of mobile ECG devices available in the market for use at home. We have summarized the usability, technical, and clinical characteristics that could allow selection of an ECG device for patients and home use. Devices available in the market are mainly intended for the detection of arrhythmias, more specifically AF, but no devices are intended for the detection of cardiac ischemic disorders. We showed that this is due to the capabilities of the devices, such as the limited measurement areas, limited number of electrodes, and recording capabilities. Clinical research concerning the devices has been primarily focused on rhythm disorders, with few studies focusing on other heart disorders, involving small test populations. Trends in the development of mobile ECG devices are inclusion of other sensors on ECG devices to increase cardiac information collected by them and a movement toward the inclusion of embedded algorithms, allowing the diagnosing of rhythm disorders.

## Abbreviations

AF: atrial fibrillation
CE: Conformité Européenne
ECG: electrocardiogram
FDA: Food and Drug Administration
fQRS: fragmented QRS
IEC: International Electrotechnical Commission
IP: ingress protection
LSB: least significant bit
MeSH: medical subject headings
STEMI: ST-segment elevation myocardial infarction
